# Myasthenia Gravis Presenting as Graft versus Host Disease after Allogeneic Blood Stem Cell Transplant

**DOI:** 10.1155/2018/7296930

**Published:** 2018-07-17

**Authors:** Zarir Ahmed, Martin Schoen, Nabeel Rajeh

**Affiliations:** ^1^Department of Internal Medicine, Saint Louis University School of Medicine, St. Louis, MO, USA; ^2^Department of Hematology and Oncology, Saint Louis University School of Medicine, St. Louis, MO, USA

## Abstract

Myasthenia gravis is a very rare manifestation of graft versus host disease after stem cell transplants. Herein, we describe a case of new-onset myasthenia gravis after a stem cell transplant 34 months ago in a patient with myelodysplastic syndrome.

## 1. Introduction

Myasthenia gravis (MG) is an autoimmune neuromuscular disorder with antibodies against nicotinic acetylcholine receptors in the neuromuscular junction. Therefore, nerve impulses are prevented from activating muscle contraction, clinically causing fatigue from repetitive skeletal muscle use. MG represents less than 1% of all complications of graft versus host disease (GVHD) after stem cell transplants (SCT) [1]. Herein, we describe a case of MG occurring after SCT.

## 2. Case

A 70-year-old male with myelodysplastic syndrome treated with double cord allogeneic blood stem cell transplant 34 months ago complicated with chronic GVHD-related glomerular nephropathy, adrenal insufficiency, and end-stage renal disease on hemodialysis presented to clinic after 2 weeks of joint pain. Physical exam revealed normal strength, and he was treated with nonsteroidal anti-inflammatory therapy. Four days later, he developed worsening joint pain, lower extremity calf pain, and hoarse voice. Examination at this time was notable for 3/5 strength in his lower extremities. He was admitted to the hospital and treated with 50 mg intravenous hydrocortisone every 12 hours for three days and intravenous fluids for a suspected postviral myositis. His symptoms resolved, and he was discharged home.

**Figure 1:**
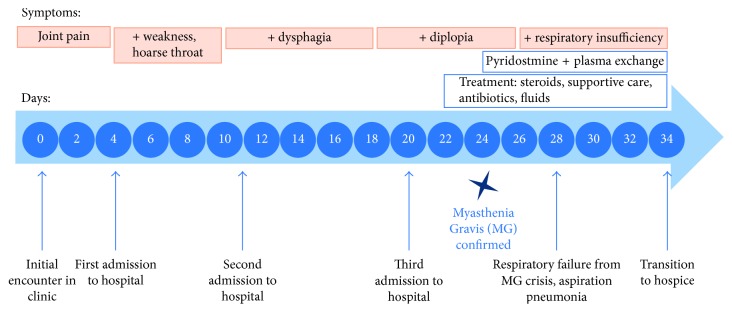
Hospital course.

Five days afterwards, he was readmitted to a different hospital for dysphagia and concern of aspiration pneumonia. A gastric tube was placed, he was treated with intravenous ceftriaxone and metronidazole, and he was sent to a rehabilitation facility. Of note, he was not treated with a quinolone or aminoglycoside antibiotic. Another four days later, he presented to our hospital with worsening pneumonia. At this time, physical exam revealed decreased proximal muscle and grip strength, weak palate elevation, diplopia upon prolonged upward gaze, and 3/5 strength in lower extremities. He did not exhibit muscle fatigue from repetitive use. Initial evaluation for this patient was unremarkable for brain or cranial nerve lesions, motor neuron disease, neuromuscular junction disorders (NMJ), or other myopathies (Table 1). He was treated with stress-dose steroids (50 mg methylprednisolone every six hours) for concern of myositis, antibiotics, and other supportive measures. Four days after this admission, the antiacetylcholine receptor (AChR) antibody (Ab) panel revealed elevated ACR binding and modulating antibodies correlating with diagnosis of myasthenia gravis. Titers for AChR binding Ab were elevated at 2.15 nmol/L, and AChR modulating Ab was elevated at 45%. He was started on pyridostigmine and plasma exchange (5 exchanges, 3500 ml per exchange). Three days into this therapy, he developed respiratory failure from MG crisis and worsening aspiration pneumonia. Due to worsening symptoms, the patient requested hospice care, and he patient passed away soon thereafter (Figure 1).

## 3. Discussion

MG is a rare late subtype of GVHD after SCT. The cause of development of MG as GVHD seems related to the presence of B lymphocytes and autoimmune antibodies [1]. Risks associated with having MG after transplantation include tapering of immunosuppression and comanifestations of chronic GVHD (especially skin involvement, glomerulonephritis, and polymyositis) [2]. A study by Heidarzadeh et al. reported twenty-three cases of MG after transplant, of which nineteen cases were associated with chronic GVHD [3]. Furthermore, they found that immunosuppressant treatment from after SCT may also increase risk of having MG [3]. Of note, our patient was previously on tacrolimus, cyclosporine, and hydrocortisone which may have contributed to his exposure.

In posttransplant patients, clinical features of MG are similar to those in nontransplant patients. Most symptoms develop between 22 and 60 months after transplantation [4]. Idiopathic myasthenia gravis typically manifests as proximal muscle weakness including diplopia. More than 50 percent of patients initially presents with ocular symptoms of ptosis and diplopia, whereas only 15 percent present with bulbar symptoms of dysarthria and dysphagia [5]. Less than 5 percent present with only limb weakness [6]. Majority of patients have persistent symptom manifestations months to years after disease diagnosis [5]. A multicenter retrospective study revealed that the maximum worsening of symptoms occurred in 77% of patients in 3 years [7].

Therapy includes acetylcholinesterase enzyme inhibition with pyridostigmine and immunosuppression with glucocorticoids or other agents such as azathioprine, mycophenolate, or cyclosporine [8]. In severe MG, plasmapheresis or intravenous immunoglobulin (IVIG) therapy can be life saving [9]. Most patients with severe MG manifestations improve after IVIG or plasmapheresis [10].

Our patient presented 34 months after hematopoietic cell transplantation and had multiple risk factors including a history of chronic GVHD that increased his risk to acquire MG. Unlike many patients who present with MG, our patient presented with atypical symptoms of joint pain rather than ocular or bulbar disturbances. This likely delayed appropriate diagnosis and treatment for MG. Furthermore, the rapid progression and severity of his symptoms within 4 to 5 weeks rather than 2 to 3 years in the average MG population is also atypical. This case warrants discussion regarding the trigger and manifestation of his disease and how it evolved so rapidly. It also serves as a reminder to keep a broad differesntial diagnosis and includes MG for posttransplant patients presenting with neurologic manifestations. Based on the National Institute of Health 2014 criteria in chronic GVHD, other diagnoses to consider in these patients include myositis, polymyositis, and myasthenia gravis [11]. In addition, immune neuropathies such as Guillain–Barré syndrome, vasculitides, demyelinating diseases such as multiple sclerosis, and encephalitis are also important considerations [8]. Appropriate diagnosis would facilitate therapy and thus potentially reverse the effects of MG and prevent adverse outcomes.

## Figures and Tables

**Table 1:** Diagnostic testing.

MRI brain	No acute cerebral infarction

Cerebrospinal fluid	Glucose 54, RBC 21, protein 25, WBC 7, 10% segmented neutrophils

Autoimmune, rheumatologic: anti Jo -1, RNP, SRP, PL-12, PL-7, PM-SCL 100, PM-SCL 75, RO-52, JO-1, antimyeloperoxidase, ANA, C-ANCA, P-ANCA, rheumatoid factor	Negative

Infectious: parvovirus B19, hepatitis A, hepatitis B, hepatitis C, EBV, west nile, CMV	Negative

Electromyography studies	Severe axonal neuropathy or demyelination. No evidence of neuromuscular junction disorder or myopathy

Acetylcholine receptor binding antibody	Elevated, 2.15 nmol/L (normal: 0–0.24)
Acetylcholine receptor modulating antibody AChR binding Ab	45% (normal: 0–20%)
